# Morphological Estimation of Cellularity on Neo-Adjuvant Treated Breast Cancer Histological Images

**DOI:** 10.3390/jimaging6100101

**Published:** 2020-09-27

**Authors:** Mauricio Alberto Ortega-Ruiz, Cefa Karabağ, Victor García Garduño, Constantino Carlos Reyes-Aldasoro

**Affiliations:** 1 Universidad del Valle de México, Departamento de Ingeniería, Campus Coyoacán, Ciudad de México 04910, Mexico; 2 Department of Electrical & Electronic Engineering, School of Mathematics, Computer Science and Engineering, City, University of London, London EC1V 0HB, UK; cefa.karabag.1@city.ac.uk; 3 Departamento de Ingeniería en Telecomunicaciones, Facultad de Ingeniería, Universidad Nacional Autónoma de México, Av. Universidad 3000, Ciudad Universitaria, Coyoacán, Ciudad de México 04510, Mexico; france@marconi.fi-b.unam.mx; 4 giCentre, Department of Computer Science, School of Mathematics, Computer Science and Engineering, City, University of London, London EC1V 0HB, UK

**Keywords:** neo-adjuvant treatment, digital pathology, tumour cellularity, machine learning

## Abstract

This paper describes a methodology that extracts key morphological features from histological breast cancer images in order to automatically assess Tumour Cellularity (TC) in Neo-Adjuvant treatment (NAT) patients. The response to NAT gives information on therapy efficacy and it is measured by the residual cancer burden index, which is composed of two metrics: TC and the assessment of lymph nodes. The data consist of whole slide images (WSIs) of breast tissue stained with Hematoxylin and Eosin (H&E) released in the 2019 SPIE Breast Challenge. The methodology proposed is based on traditional computer vision methods (K-means, watershed segmentation, Otsu’s binarisation, and morphological operations), implementing colour separation, segmentation, and feature extraction. Correlation between morphological features and the residual TC after a NAT treatment was examined. Linear regression and statistical methods were used and twenty-two key morphological parameters from the nuclei, epithelial region, and the full image were extracted. Subsequently, an automated TC assessment that was based on Machine Learning (ML) algorithms was implemented and trained with only selected key parameters. The methodology was validated with the score assigned by two pathologists through the intra-class correlation coefficient (ICC). The selection of key morphological parameters improved the results reported over other ML methodologies and it was very close to deep learning methodologies. These results are encouraging, as a traditionally-trained ML algorithm can be useful when limited training data are available preventing the use of deep learning approaches.

## 1. Introduction

Digital pathology has recently become a major player in Cancer research, disease detection, classification, and even in outcome prognosis [1,2,3,4]. Perhaps the most common imaging technique is Hematoxylin and Eosin (H&E), where H stains nuclei blue and E stains cytoplasm pink [5]. Additionally, other immunohistochemistry methods (IHC) that use antibodies to stain antigens or proteins in the tissue are more specific and they can complement H&E [6]. For instance, the cluster of differentiation 31 (CD31), which is commonly found on endothelial cells, is used as an indication of the growth of blood vessels in tumours, or angiogenesis [7,8,9]. CD34 is found on hematopoietic cells, mesenchymal stem cells, and it is required in certain processes, like infiltration of eosinophils into the colon [10], or the dendritic cell trafficking and pathology in pneumonitis [11], Ki67 is normally associated with cell proliferation and it can be used as a prognostic factor for gliomas [12], breast cancer [13], and colorectal adenocarcinomas [14]. Computer-assisted diagnosis (CAD) [2] is based on the quantitative analysis to grade the level of the disease, but, recently, other clinical-pathological relationships with the data have been explored [15,16]. For example, a better understanding of mechanisms of the disease evolution process [1] and even prognosis information [3].

Breast Cancer is a common disease both in terms of incidence and deaths, with approximately 252,710 new cases and 40,610 deaths in 2017 in the United States alone [17]. In 2018, 30% of new cases Cancer among females cases in the US were breast cancer, which placed it the second place in mortality [18]. Numerous imaging analysis methods are employed in order to study these cancers [19]. Common treatments for breast cancer are surgery, radiation, chemotherapy, or targeted therapy. Breast cancer neo-adjuvant treatment (NAT) is a therapy for advanced cases that provides useful information for breast-conserving surgery [20]. NAT provides prognostic and survival information [21] as well as a rate of local recurrence [22]. The efficacy of NAT is determined by means of the pathological complete response (pCR) and this can be assessed by the Residual Cancer Burden (RCB) [23]. RCB is supported by two metrics: residual Tumour Cellularity (TC) within the Tumour Bed (TB) and the assessment of lymph nodes. RCB is scored in a continuous value, but it is further categorised in four classes RCB-0 to RCB-III. Subsequently, TC, which is defined as the fraction of malignant cells within the image patch, is a key parameter for RCB computation [24,25]. Currently, TC is manually assessed by an eye-balling routine estimating the proportion of TB and this procedure is time-consuming and requires an experienced pathologist.

Neo-adjuvant treatment (NAT) chemotherapy refers to a treatment that is administrated before Cancer surgery [26,27,28]. The first successful results of NAT chemotherapy were demonstrated in the 1980s [29,30]. Some of the benefits of NAT are: tumour size reduction, better prognostics, and, even in some cases, surgery can be avoided [31]. In addition, patients with large tumours could be eligible for breast-conserving or a less tumour size surgery extraction.

TC assessment problem was first addressed by a hand engineering approach based on nuclei segmentation and feature extraction. In the first step, nuclei segmentation needs to be implemented [1]. This is a challenging task and some common techniques are based on active contours [32], watersheds [33], or graph cuts [34], which are either designed for nuclei [33] or lymphocytes segmentation. [32] Based on the segmentation application, speed, accuracy, or automation level might be required [34]. In the present study, automated segmentation is performed and, as it has been reported in some tissue classification studies, accuracy nuclei segmentation does not guarantee better outcome assessment between benign and malignant tissue [2]. When processing a high number of image files, speed constraint is preferable. After segmentation, many features can be extracted, for instance, cell shape, size, and texture [33,34,35], and also, features from regional and the global image can provide valuable information [36]. These parameters can be used for diagnostic purposes to classify tissue malignancy [36,37,38,39] and also for grade disease level assignment. Ref. [33] For instance, a study conducted by Dong and co-authors [40] categorised intraductal lesions in breast cancer by an adequate feature extraction and machine learning (ML) classification.

ML algorithms are trained to learn from the parameters extracted [15]. Some supervised algorithms are Support Vector Machines [41], Boosted Trees [42], and K-Nearest Neighbours [43]. Besides diagnosis, different new clinicopathological relationships with features have been discovered, for example, [16] revealed the relation between stroma morphology and prognosis of breast cancer patients and [44] studied quantification and distribution of tumour-infiltrating lymphocytes (TILs) as prognostic and predictive biomarker.These type of digital pathology methodologies can also useful for TC assessment. A full hand-engineering method for this task was proposed by Peikari [45]. This methodology was based on the extraction of a vector with 125 parameters [45].

Separate to traditional ML techniques, another approach to estimate TC is by Deep Learning (DL) techniques. In the last decade, there has been increased interest in DL methods. One of the main architectures or models is based on the use of neural networks, and a particular case is a Convolutional Neural Network (CNN) [46]. The main advantage of DL techniques is that they do not require the extraction of features manually or by training, as the network learns a series of parameters and weights by itself. However, to achieve this, the network requires a relatively large number of training images with labels, and the number of training data can impact on the performance. In many histopathology applications, labelled training data are still limited as compared to other imaging applications, such as everyday images, like cats and dogs. With the spread use of whole slide digital scanners [47], numerous histopathology images have become available for research purposes. Some have been released to the research community in general in the form of challenges (e.g., https://grand-challenge.org/challenges/) in order to encourage research groups globally to work together, gather annotations, provide training, and testing data sets and benchmark algorithms. The present work follows the 2019 SPIE-AAPM-NCI BreastPathQ: Cancer Cellularity Challenge with the specific objective of “development of quantitative biomarkers for the determination of cancer cellularity from whole slide images (WSI) of breast cancer hematoxylin and eosin (H&E) stained pathological slides” (https://breastpathq.grand-challenge.org/) and addresses the development of quantitative biomarkers for the challenge.

CNNs have been used for different Histopathology tasks, like segmentation [48,49], detection of a specific image properties [50], and image grade classification [38,51]. Breast cancer tumour cellularity has also been addressed by deep techniques. For instance, Ziang Pei [52] implemented a direct method based on deep and transfer learning approach with the advantage of avoiding cell segmentation. Akbar [20] presented a traditional hand-engineering approach and a deep neural network.

The methodology in this study is based on a hand-engineering approach similar to the one that was described by [45] based on a 125 parameter vector size, but we selected 22 parameters after a correlation analysis of extracted features with TC. Additionally, the methodology described here is similar to Dong et al. parameters from nuclei; we also include parameters from the neighbourhood of the nuclei and from the full image patch. The study by Fondon [36] also analysed regions around the nuclei; however, our study includes morphology parameters from whole breast ducts region. Thus, the main contributions of this paper in the methodology are the segmentation algorithm that is based on the enhancement of the nuclei region, and an algorithm for breast ducts detection and the parameter derivation and selection and its correlation to tumour cellularity. In the results, the correlation analysis of the morphological parameters with cellularity revealed that stroma concentration has the strongest correlation with TC, which is in agreement with the results that were presented by [16]. Finally, the results were validated with the ICC and compared with similar studies [20,45] indicating an increase in ICC. The methodology described only requires a reduced set of training parameters. Therefore, the methodology described in this paper improves previous results and it may be useful in cases when large training data sets, which are normally required for deep learning approaches, are not available.

## 2. Materials and Methods

### 2.1. Materials

**Figure 1:**
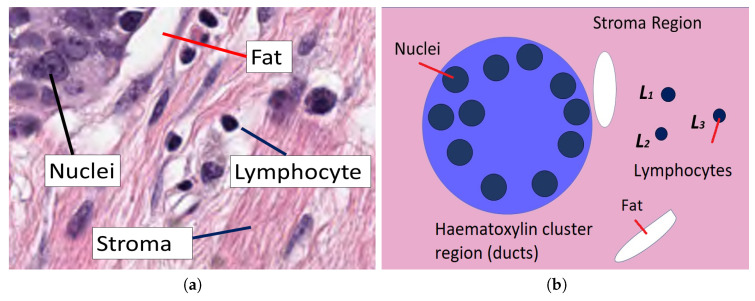
(**a**) Stroma region which shows several clusters, regions of fat as well as some lymphocytes. (**b**) Graphical description of the elements that are analysed in this work. Within a stroma, the connective tissue shown in pink region, ducts appear as clusters stained with haematoxylin and contain several nuclei. Outside these ducts, regions of fat appear white and lymphocytes appear purple.

The data set used in this work consists of 2579 patches of tissue stained with H&E, which were extracted from 64 different patients under Neo-adjuvant Treatment. The size of each patch is 512×512 pixels. As a reference, a breast tissue is formed of a connective tissue, named stroma, as seen in pink. Lymphocytes can be seen as dark blue round objects and fat zones as white areas. It also contains ducts, which are responsible for carrying milk, and sometimes arteries, which can be seen as regular clusters of darker blue nuclei grouped into the region (Figure 1).

The residual cellularity of each patch was evaluated by 2 pathologists and this assessment was considered to be the ground truth (GT) for this study. The data was released as part of the challenge 2019 and it was collected at the Sunnybrook Health Sciences Centre, Toronto. It comprises a set of whole slide images (WSIs) that have been stained with H&E [45] from 64 patients with residual invasive breast cancer on re-section specimens following NAT therapy. The specimens were handled according to routine clinical protocols and WSIs were scanned at 20× magnification (0.5μm/pixel).

**Figure 2:**
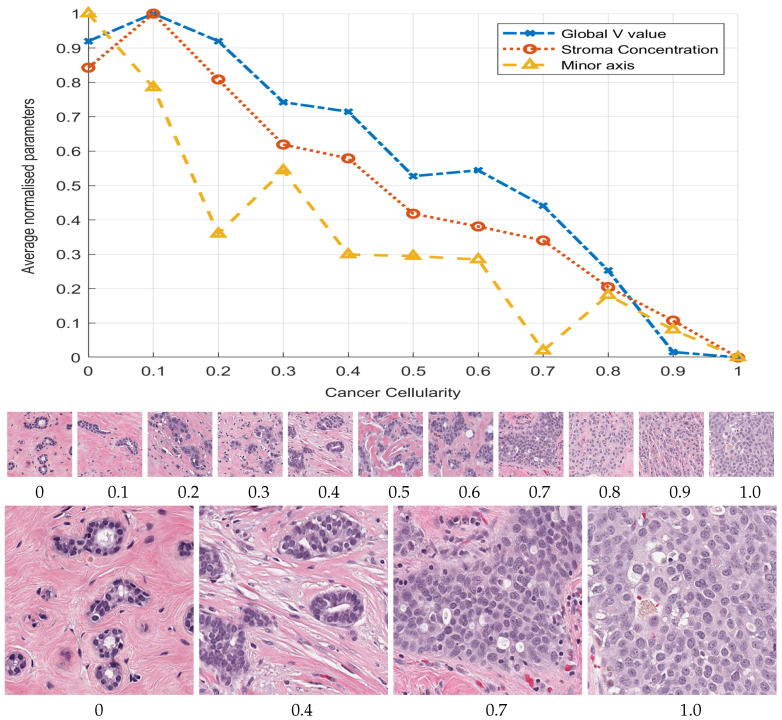
Graphical display of three selected morphological parameters against the cellularity: Global stroma filtered region (o), minor axis (△) and Value concentration in region (x). Cellularity was manually ranked by a pathologist and the parameters automatically extracted. The plots represent average parameter value normalised with maximum value to be compared in the same graph. The graph indicates an inverse relationship between respective parameter and cellularity. Thumbnails show images with cellularity values from zero to one and magnified versions of cases with values 0, 0.4, 0.7, and 1, where the prevalence of cancerous cells can be clearly observed.

The images were divided into a training and validation set of images. The cellularity value distribution for the whole data-set is uneven, i.e., most of the patches correspond to cellularity zero and fewer patches were available for higher cellularity. The training images were selected uniformly distributed from cellularity zero to one in order to have an even amount of benign and malignant nuclei. First, 212 images with selected cellularity values from 0 to 1 were processed and 4533 nuclei cells from those selected images were extracted and its corresponding features computed and used as a training set. The remaining 2367 patches were used for validation. Figure 2 shows the selected patches with different levels of cellularity. A third set of test data of 1119 images was available for the challenge contest. These three sets were used in this work.

### 2.2. Methodology

**Figure 3:**
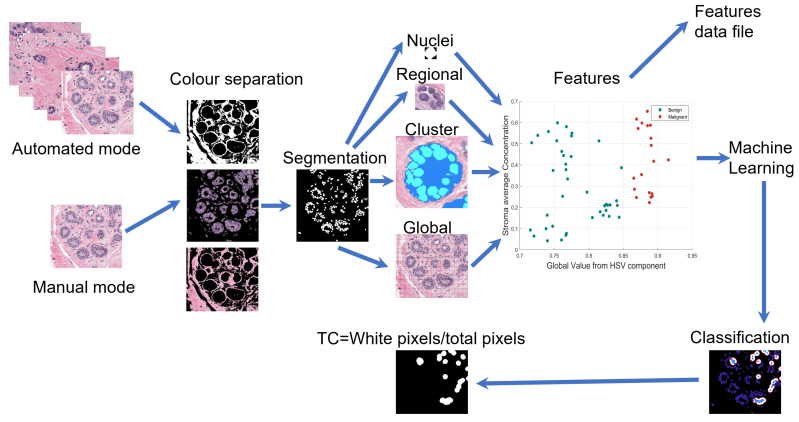
Graphical illustration of the pipeline of the methodology. Under two operational modes images are processed to extract features at nuclei, regional, cluster, and global image regions either to classify and assign Cellularity or to extract same features in order to an archive output.

The proposed methodology consists of a sequence of traditional image processing routines, computer vision, and machine learning algorithms that automatically process the full validation set. A large amount of morphological parameters can be extracted and stored orderly in an output table file. A master control routine is responsible for selecting one by one the corresponding image patch to be processed. There are two operational modes. A manualmode useful to train machine learning algorithms. In this mode, nuclei from the selected image with a known classification assignment are fed to the algorithm and output features are saved in an output data file. Subsequently, an automated mode processes the full validation set and gives a TC estimation. This mode is able to process thousands of patch images. Figure 3 presents this process in a diagram.

The methodology was implemented in Matlab^®^ (The Mathworks™, Natick, MA, USA) 2019b version, with functions from the digital image processing, statistical, and machine learning toolboxes. Additionally, QuPath [53], an open source software for digital pathology image analysis, was used to validate the segmentation results obtained by the methodology. Three regions of interest were selected from each image patch, and more than 150 morphological parameters were extracted at inner segmented cell region, neighbourhood around segmented cell, and the full image patch.

### 2.3. Segmentation of Nuclei

**Figure 4:**
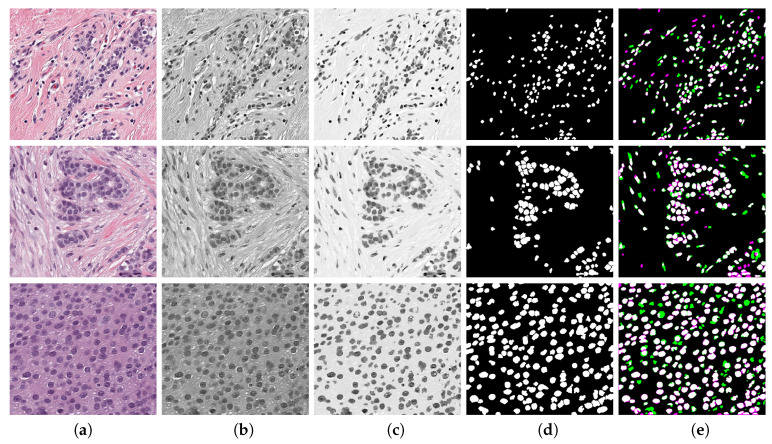
Enhancement of nuclei area. First row corresponds to a Tumour Cellularity (TC) value of zero, second row is an example of TC =0.5 and third row is TC =1. Images in column (**a**) are the original image, (**b**) gray level images, (**c**) enhanced images, notice that the nuclei region is darker whilst background becomes lighter, (**d**) Binary image obtained with an adaptive threshold value estimated directly from strongest correlated parameter, and (**e**) validation of nuclei segmentation by Jaccard index. Reference image was manually segmented by using QuPath Platform.

At the nuclei region, cells from the image patch are segmented by colour separation and binarisation. The image is converted to HSV colour space and, by K-means clustering [54], three main colour images are extracted, say Ip for pink, Ib for blue, and Iba for the remaining background component image. A special procedure enhances Ib and weakens background region is calculated as:

(1) $$In=K_{1}\left(Io.*Im\right)+K_{2}Io+\gamma \left(Im,0.1\right)$$

where Io is the RGB image converted to gray levels and Im is gray image after a median filter of size 3×3 was applied. γ is the gamma correction Im of parameter 0.1 represented as γ(Im,0.1). This value was selected as low to start from a lighter background. $K_{1}$ and $K_{2}$ are constants that control enhancement of nuclei and weaken background intensity, respectively, and they were experimentally adjusted. First, both of the constants were fixed to 0.5 and, as $K_{1}$ is increased and $K_{2}$ is reduced, the nuclei region is enhanced. The enhanced image In is binarised by Otsu’s algorithm [55] combined with watershed [56] separation of touching nuclei. This segmentation procedure is seen in Figure 4 for three TC cases. A validation analysis of this procedure was done by means of Jaccard Index [57], as shown in (e). The reference images were obtained by a manual segmentation while using the QuPath platform. The results indicate an average Jaccard index of 0.73 after comparing cases of low, medium, and high TC. Although this result might be improved, Peikari and co-authors [45] suggest that cell segmentation accuracy does not have a significant effect in TC assessment.

### 2.4. Extraction of Morphological Parameters

Using the binary image, a set of morphological parameters has been obtained: area, perimeter, roundness, eccentricity, centroid X, centroid Y, major axis, minor axis, and orientation angle. Additionally, a sub region inside the segmented cell body was determined in order to compute the texture and mean HSV values of the nuclei cell body.

**Figure 5:**
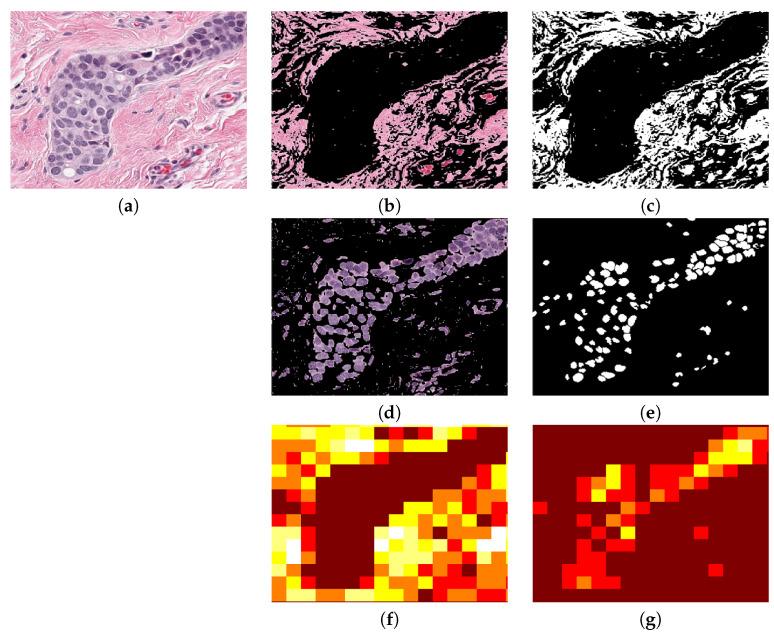
Regional analysis of the data. (**a**) Original image. (**b**) Ip, stroma region image obtained from colour separation. (**c**) Binarised Ip image. (**d**) Ib nuclei image obtained from colour separation. (**e**) Binarised Ib image. (**f**) Regional image concentration of Ip in which brown colour indicates the lowest and white is the highest region concentration. (**g**) Regional density concentration of Ib. This example was processed at 30×30 pixels window.

**Figure 6:**
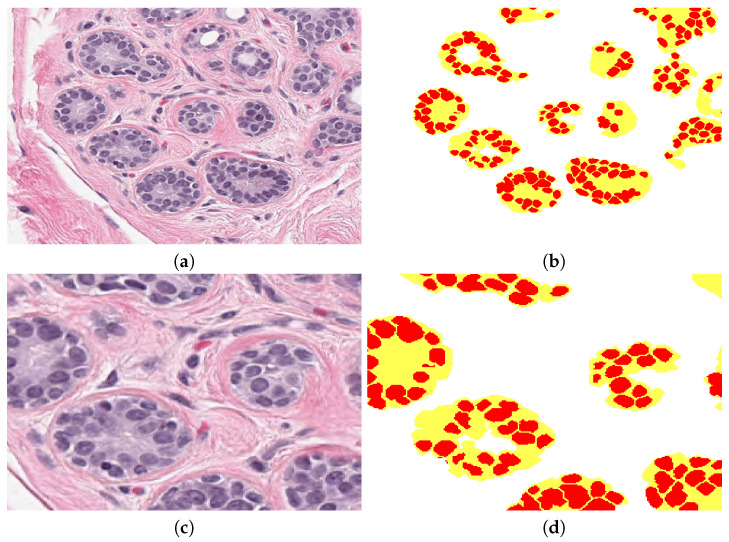
(**a**) The original patch with clear breast ducts. (**b**) Clusters detected by the methodology, every duct or cluster is in yellow and cells inside are in red. Background is labelled in white. (**c**,**d**) Magnified regions of (**a**,**b**).

The parameters were extracted from different window sizes surrounding the segmented cell: 30×30, 60×60, 90×90, and 120×120 pixels. The statistical analysis indicated that the best correlation was obtained from the smallest window size. The parameters estimated are the four bins histogram from HSV image and also the regional concentration *R* of Ip, Iba, and Ib from its binary image, determined by the ratio of total white pixels $T_{W}$ by total pixels $T_{P}$ inside window neighbourhood region, $R=T_{W}/T_{P}$. Figure 5 shows a sample patch image, with its corresponding pink, blue, binary, and concentration image components. Brown areas indicate low concentration and white and yellow areas are high concentration. Breast normal tissue images present ducts and sometimes arteries, which are clearly seen as regular clusters of darker blue nuclei grouped into the region, see Figure 6. These are detected based on set theory: let U be the full image, D be the cluster or duct, D⊆U ≠∅. Subsequently, D regions are obtained by subtracting background image Iba from original image U. The following morphological parameters can be extracted from these regions: total cluster area, roundness, number of cells inside cluster, and distance from cells centroid to cluster centroid.

Concentration parameters from the complete full image patch were computed using original RGB image and transformed to HSV. Additionally, a pink colour filter was implemented by means of the Matlab^®^ colour thresholder, which extracts stroma region from the image and computes its mean value. A summary of the full set of features extracted is presented in Table 1; notice that regional parameters are computed at the different window sizes.

### 2.5. Correlation Analysis of Morphological Parameters to TC

The full training set was processed to determine all of the morphological parameters at the three region of interest and those corresponding to Cellularity values equal to (0,0.1,0.2,…,1.0) were selected, and their mean, standard deviation, maximum, and minimum values were computed. Subsequently, a linear regression and lasso analysis were determined from both analyses. Parameters of coefficient above 0.80 from linear regression and in concordance with lasso selected parameters after redundant removal yield 22 parameters that have the strongest correlation with TC. The plot in in Figure 2 illustrates three parameters with the strongest correlation with TC.

**Figure 7:**
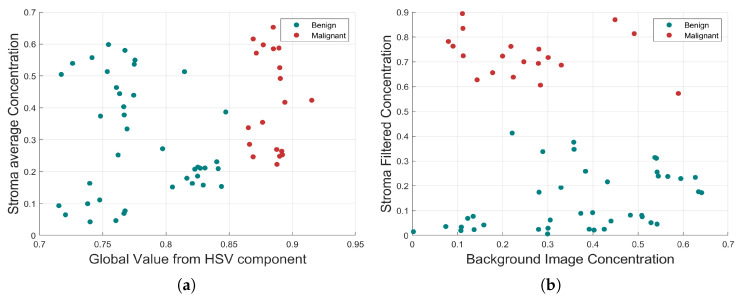
Morphological relationship between the four strongest correlated parameters. (**a**) Global value from HSV component and Stroma average Concentration. (**b**) Background Image Concentration and Stroma Filtered Concentration. In both cases, the benign and malignant cells are highlighted with different colours, which indicate a clear separation.

First, the morphological parameters related to segmented nuclei are: eccentricity, roundness, major axis, minor axis, and perimeter. Subsequently, parameters computed at neighbourhood region are: nuclei density concentration, Hue, Value, and Saturation histograms of the regional HSV colour map image. Finally, parameters that are computed from the full image are: HSV average values from HSV components, nuclei concentration, basement concentration, and stroma average concentration determined at output of the pink colour filter. Some of these parameters are graphically displayed in Figure 7.

### 2.6. Training of Machine Learning Algorithms

**Figure 8:**
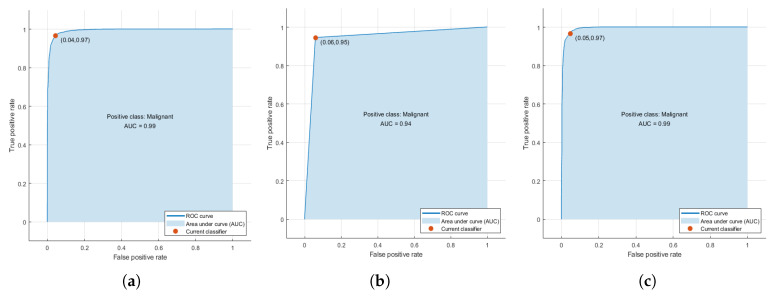
(**a**) Receiver operating characteristic (ROC) curves obtained during training phase for the three selected algorithms. (**a**) Support Vector Machines (SVM), (**b**) AdaBoost, and (**c**) K-Nearest Neighbour (KNN). The higher accuracy value achieved is obtained only with the training set that is highly correlated with Tumour Cellularity (TC).

Machine learning algorithms were trained with the parameter vector of size 22 determined by the statistical correlation analysis. From the training set, 4533 segmented cells were processed and its corresponding extracted parameters were used to generate a prediction function that classifies nuclei cells between malignant and normal cells. Three algorithms were tested: Support Vector Machines, Nearest K-Network, and AdaBoost. The accuracy of training process for every selected method with the training data showed values up to 0.99 due to a high correlation selected features used for training. TPR achieved are 0.97 for SVM, 0.95 for AdaBoost, and 0.97 for KNN (Figure 8). Support Vector Machines (SVM) is a training algorithm for optimal margin classifier [41], and it is based on a determination of a decision function of pattern vectors *x* of dimension *n* classifying in either A or B, in our case benign or malignant cells. The input is a set of *p* examples of $x_{i}$, i.e., the 22 strongest correlated features extracted. K-Nearest Neighbour method [42] was also selected, because it is one of the most well known algorithms within clustering and data classification, in our case between benign and malignant classes. AdaBoost [43] is a decision tree type learning algorithm that starts from observations of a certain item that is represented by branches and goes to conclusions about item target value or leaves. It has a best performance on binary classification problems.

### 2.7. Assessment of Tumour Cellularity

**Figure 9:**
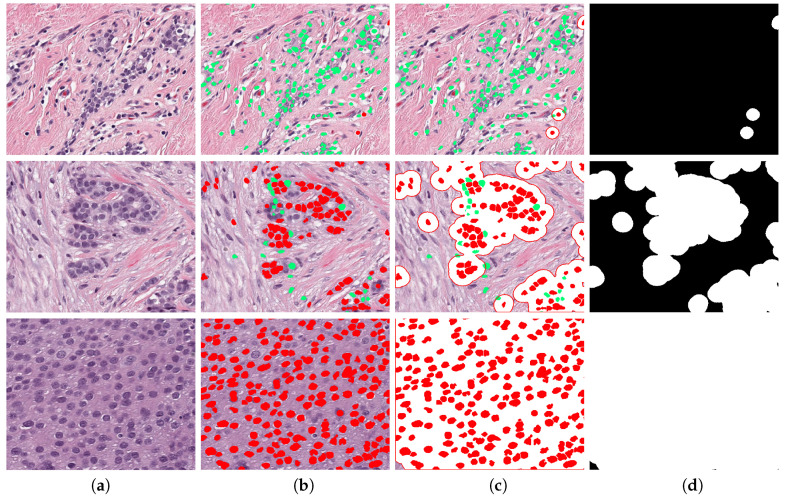
Visual description of the method. Three TC cases are presented in each row: 0, 0.5 and 1. (**a**) The original image, the image is segmented and key parameters are computed, then a classification predictor estimates either malignant or benign cells, shown in red and green, respectively in (**b**). A dilation of segmented malignant nuclei estimates full cytoplasm of every detected malignant cell (**c**) and TB region is shown in white in (**d**). The cellularity metrics calculated by the proposed methodology are: TC=0.0113, TC=0.5181 and TC=0.9936.

The methodology to estimate TC is illustrated in Figure 9, with three representative images with increasing TC from top to bottom in each row. In Figure 9a the nuclei are segmented and their corresponding parameters are extracted. Subsequently, the prediction function classifies every cell in either benign (green) or malignant (red), as illustrated in Figure 9b. Next, an estimation of full cell cytoplasm is done by morphological dilation drawn as the white circles around malignant cells (Figure 9c). The full cellularity that is detected region is shown in Figure 9d. TC is computed as the ratio of the area that is covered by cellularity (white in the figure) over the total area (white and black in figure).

## 3. Results

**Figure 10:**
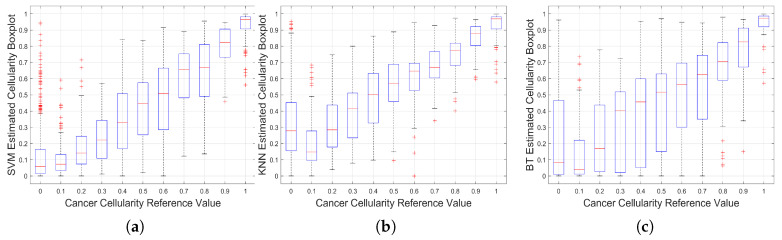
Results of implementation on Training Data. Boxplots for the Cancer Cellularity Reference Value against: (**a**) SVM estimation, (**b**) KNN estimation and (**c**) BT Estimation. It should be noted that large boxplots correspond to large variations of the estimations and as such, SVM shows the lowest variability.

An automated estimation of TC was computed from two test data sets. Three prediction functions that were trained by machine learning algorithms were determined to be used with the automated processing software of breast cancer images that classifies cells and computes TC. The method was tested with a training set of 2579 images that were already classified by a pathologist with a TC value. Additionally, it was tested with the 1119 images for submission of SPIE Breast Challenge, with an unknown TC value. Figure 10 shows the statistical behaviour of the method’s result for the training set as boxplots.

Dispersion plot indicates the method for approximating to the pathologist classification assignment. The results have a better approximation at higher cellularity values (TC>0.70) and performs well with KNN algorithm. Additionally, around the middle region (0.4<TC<0.6) has a good approximation with AdaBoost. At low cellularity values (TC<0.3), three methods present deviation, with its higher at cellularity zero, which correspond to images with only benign nuclei cells. According to Minimum Square Error (MSE), SVM performs better overall in the cellularity region. This result can be validated by a visual inspection of boxplots of Figure 10 in the three cases there is a positive correlation between the actual cellularity (horizontal) and the estimated cellularity (vertical). However, SVM shows less dispersion, especially in the lower values of cellularity as compared with the other two techniques.

**Figure 11:**
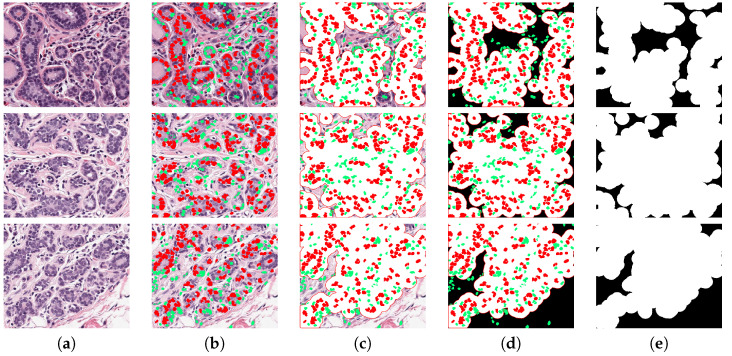
Three cases where the TC was incorrectly calculated. Each row corresponds to a patch with incorrect TC. Column (**a**) illustrates the original image, and columns (**b**–**e**) show the step by step process to assess cellularity. (**b**) Corresponds to the segmented image and classified into benign (green) and malignant (red). Columns (**c**,**d**) show expanded region of malignant cells. Column (**e**) corresponds to cancer cell region in white, used to compute TC. Three worst cases correspond to a TC of zero; this means there would not be any malignant cell and TC image must be completely black. Several cells were miss classified which yields to a TC wrong assessment. Estimated TC values are: 0.75, 0.81, and 0.75, instead of zero.

In order to analyse the limitations of the algorithm, the TC assessment outcomes with the highest errors were analysed. Figure 11 presents three cases where the TC was incorrectly calculated. All three cases in the figure are of benignant tissue with no cellularity; this means that most of the segmented cells should be marked as benignant (green), but several of them are shown in red (b–d), which corresponds to false-positive cases. The expanded area around the cell (e) yields a high TC value instead of the correct value which should have been zero. We assume that this problem is because of the limited amount of cells used to train the algorithm. This problem also explains some of the outliers on the boxplots presented in Figure 10, which are mainly observed at low cellularity. Additionally, this suggests that other classification algorithms should be evaluated.

Statistical analysis of the training set revealed 22 key parameters that have a strong correlation to TC. The Stroma concentration (r =−0.9786), global Value of HSV component (r =−0.9728), regional histogram bins (r =−0.9659), and minor axis (r =0.8939) from nuclei morphology were the strongest parameters, as shown in Figure 2.

This result revealed that the stroma region has a significant relation to TC, which is in agreement with the results of the hand engineering method by [45] that was trained by a 125-dimensional feature vector reported a 0.75 ICC (first column of Table 2). Lower and upper-bounds are shown in square brackets. Our methodology is also a hand engineering, but trained with only 22 key morphological parameters (second column of Table 2), indicates a 0.78 ICC. This result outperformed those that were obtained by the method of Peikari. The results based on Deep Learning techniques like a combined hand engineering Deep Neural Network reported by Akbar [20] is slightly above the proposed methodology (third column of Table 2). Finally, the methodology proposed was used to process the test set and the results were submitted to the challenge contest. The prediction probability result obtained from contest was $P_{k}=0.76$.

## 4. Discussion

A computer methodology that automatically processes H&E histopathology digital images based in the extraction of main morphological parameters at a cell, regional, and global level is presented in this paper. The methodology processed a training set of breast cancer images under NAT treatment and the results indicate 22 key morphological parameters are strongly correlated with cellularity. Interesting results were revealed from the correlation analysis of the morphological parameters. The strongest related parameter was stroma density, in agreement with Beck et al. [16], which is, the histology of stroma correlates with prognostic in breast cancer. Three different machine learning algorithms for cell classification were evaluated and compared in order to determine tumour regions. The best result was obtained with Support Vector Machines (SVM) algorithm. The relevance of this paper is a selection of a key parameters to train the algorithms, which results in a better performance of similar techniques; however, the reported deep learning algorithms outperform this result, which is a motivation to explore these techniques in the future.

## Figures and Tables

**Table 1:** Summary of extracted features. Features at regional level are computed at four different window sizes. Features from the full image represent average values.

Nuclei	Area	Eccentricity	Roundness	Centroid x, y
	Perimeter	Orientation	Major Axis	Minor Axis
	Mean Texture Contrast 1	Mean Texture Contrast 2	Mean Texture Homogenity 1	Mean Texture Homogenity 2
	Mean H value inside nuclei	Mean V value inside nuclei	Mean S value inside nuclei	
Regional Concentrations	Stroma Ip	Background Iba	Nuclei Ib	Epithelial tissue from Ib
	Mean H in window	Mean S in window 2	Mean V in window	
	Mean intensity Histogram H 1	Mean intensity Histogram H 2	Mean intensity Histogram H 3	Mean intensity Histogram H 4
	Mean intensity Histogram S 1	Mean intensity Histogram S 2	Mean intensity Histogram S 3	Mean intensity Histogram S 4
	Mean intensity Histogram V 1	Mean intensity Histogram V 2	Mean intensity Histogram V 3	Mean intensity Histogram V 4
Clusters (ducts)	Cluster area	Cluster roundness	Cells inside cluster	Distance to centroid
Global Image Concentrations	Stroma Ip	Background Iba	Nuclei Ib	
	H value	V Value	S Value	

**Table 2:** Comparison of the Intra-class Correlation Coefficients (ICC) of the proposed methodology against a Hand Engineering Methodology (Peikari [45]) and a combined deep learning and hand engineering methodology (Akbar [20]). Lower and upper bounds are shown in square brackets. Notice the closeness of the results of the proposed methodology against the Deep Learning approach.

	Hand Engineering	Key Parameters	Combined Deep Network
	(Peikari)	(Our methodology)	(Akbar)
ICC	0.75	0.78	0.79
[L,U]	[0.71, 0.79]	[0.75, 0.80]	[0.76, 0.81]

## Abbreviations

The following abbreviations are used in this manuscript:

TC	Tumour Cellularity
NAT	Neo-Adjuvant treatment
WSIs	Whole slide images
H&E	Hematoxylin and Eosin
ICC	Intraclass correlation coefficient
ML	Machine Learning
IHC	Immunohistochemistry
CAD	Computer-assisted diagnosis
RCB	Residual Cancer Burden
pCR	Pathological complete response
TB	Tumour Bed
GT	Ground Truth
HSV	Hue, Saturation, Value
RGB	Red, Green, Blue
SVM	Support Vector Machines
